# The Association Between Vitamin D and Early Childhood Caries: A Systematic Review and Meta-Analysis

**DOI:** 10.3290/j.ohpd.b4928565

**Published:** 2024-02-02

**Authors:** Shuaiqi Ji, Kai Zhao, Lei Ma, Xiaohang Chen, Dali Zheng, Youguang Lu

**Affiliations:** a PhD Student, Fujian Key Laboratory of Oral Diseases, School and Hospital of Stomatology, Fujian Medical University, Fuzhou, China; Department of Preventive Dentistry, School and Hospital of Stomatology, Fujian Medical University, Fuzhou, China. Data collection, wrote the manuscript, reviewed the literature, read and approved the final manuscript.; b Dentist, Department of Stomatology, Dushu Lake Hospital Affiliated of Soochow University, Medical Center of Soochow University, Suzhou Dushu Lake Hospital, Suzhou, Jiangsu, China. Analysed and interpretated the data, read and approved the final manuscript.; c Professor, Department of Prosthodontics, The Affiliated Hospital of Qingdao University & School of Stomatology of Qingdao University, Qingdao, China. Analysed and interpretated the data, read and approved the final manuscript.; d PhD Student, Fujian Key Laboratory of Oral Diseases, School and Hospital of Stomatology, Fujian Medical University, Fuzhou, China; Department of Preventive Dentistry, School and Hospital of Stomatology, Fujian Medical University, Fuzhou, China. Performed the survey, quality control of the investigation, edited the manuscript, read and approved the final manuscript.; e Professor, Fujian Key Laboratory of Oral Diseases, School and Hospital of Stomatology, Fujian Medical University, Fuzhou, China; Department of Preventive Dentistry, School and Hospital of Stomatology, Fujian Medical University, Fuzhou, China. Performed the survey, quality control of the investigation, edited the manuscript, read and approved the final manuscript.; f Professor, Fujian Key Laboratory of Oral Diseases, School and Hospital of Stomatology, Fujian Medical University, Fuzhou, China; Department of Preventive Dentistry, School and Hospital of Stomatology, Fujian Medical University, Fuzhou, China. Establishment of the database, data analysis, wrote the manuscript, read and approved the final manuscript.

**Keywords:** early childhood caries, vitamin D, 25(OH)D

## Abstract

**Purpose:**

Previous surveys have reported that children with vitamin D deficiency were likely to suffer from early childhood caries (ECC). The aim of this systematic review and meta-analysis was to determine 1. whether the status of vitamin D is intrinsically related to the occurrence of ECC and 2. the optimal level of vitamin D for the prevention of ECC.

**Materials and Methods:**

The database of PubMed, Web of Science, Cochrane, Embase and Google scholar were searched for targeted literature. The eligibility criteria were observational studies in which children with ECC were compared to children without ECC in terms of their vitamin D status. Applying the Newcastle-Ottawa tool, study selection, data extraction, and risk of bias assessment were performed by 2 reviewers independently. Meta-analysis was performed using the Cochrane Collaboration’s Review Manager 5.4 software.

**Results:**

501 articles were retrieved from the electronic databases; 11 studies were finally included in systematic review, 10 studies of which were submitted to meta-analysis. The 25(OH)D levels in the ECC group were statistically significantly lower compared with that in the caries-free group (WMD = -13.96, 95% CI: [-19.88,-8.03], p < 0.001), especially in regard to the association between S-ECC and vitamin D (WMD = -18.64, 95% CI: [-20.06,-17.22], p < 0.001). The subgroup analyses in terms of geographical region demonstrated that children with a level of 25(OH)D of 50–75 nmol/l were more likely to have ECC than those with over 75 nmol/l (OR = 1.42, 95% CI: [1.26,1.60], p < 0.001), with data from Asia and Europe combined for analysis

**Conclusions:**

The level of vitamin D was lower in children with ECC than in caries-free children, and the correlation between S-ECC and vitamin D was even stronger. The optimal 25(OH)D level for preventing occurrence and development of ECC was ≥ 75 nmol/l. Thus, clinicians should view the development of early caries also from a systemic perspective.

Early childhood caries (ECC), as one of the most common diseases occurring in children aged 6 years or younger, is associated with pain and discomfort in the primary dentition; it affects the growth and development of children, seriously hindering the development of their overall health.^44^ Severe early childhood caries (S-ECC), the severe phase of the illness, refers to smooth-surface caries in children younger than 3 years of age or ≥ 1 cavitated missing teeth (due to caries), filled smooth surfaces in primary maxillary anterior teeth, or decayed, missing, or filled surfaces (dmfs) score of ≥ 4 (age 3 years), ≥ 5 (age 4 years), or ≥ 6 (age 5 years).^15^ Although the local factors causing caries in children, such as feeding, diet, household environmental and bacterial factors have been identified and discussed,^44^ there are also certain recently discovered systemic risk factors present in young children, such as iron deficiency anemia and lack of vitamin D.^3,27^

Vitamin D, a steroid hormone, is an essential nutrient that has recently received greatly increased public attention due to widespread vitamin D deficiency.^9,15,21,29^ It is well known that the occurrence of rickets in childhood is due to vitamin D deficiency.^34^ As an exogenous factor, the lack of exposure to sunlight is mainly responsible for this deficiency.^24^ The vitamin D status measured by the level of serum 25-hydroxyvitamin D, a widely accepted biomarker,^45^ may thus vary from season to season, with it being higher in summer and lower in winter.^42^ This may explain why the levels of serum 25-hydroxyvitamin D in children with ECC differ between winter and summer. The reference value of vitamin D in blood is controversial. The Institute of Medicine (USA) and the Endocrine Society (Canada) set two cut-off values: 20 ng/ml (50 nmol/l) and 30 ng/ml (75 nmol/l),^22,37^ respectively, with the former based on preventing nutritional rickets. Recently, the relationships between deficits of vitamin D and relevant oral diseases such as caries and periodontal disease have been increasingly elucidated.^9,32^ Vitamin D impacts oral health not only by influencing bone metabolism, but also by compromising tooth-germ formation. Because ameloblasts and odontoblasts are target cells for the active form of vitamin D, a lack of vitamin D during the process of odontogenesis can lead to developmental defects such as enamel hypoplasia, leaving teeth susceptible to the development of caries.^18^ A great deal of observational studies have shown that vitamin D status in childhood plays a critical role the occurrence and development of ECC.^5,13,26,38,39,42^ Nevertheless, the results are not always consistent, with some studies supporting the correlation between higher ECC rates and lower vitamin D levels and others finding no relation.^20,25,35^ Although there is a systematic review about the relation between prenatal or childhood serum levels of vitamin D and dental caries in childhood,^10^ the lack of meta-analysis (due to insufficient studies) has left us with no clearer picture of the relationship between vitamin D and ECC. In addition, the majority of studies selected by the previous systematic review concentrated on children aged from 1 to 12 years old, spanning two periods – primary dentition and mixed dentition – in which the associated factors influencing caries or vitamin D deficiency were different.^1,16^

The aim of the present study was to investigate whether the status of vitamin D is intrinsically related to the occurrence of ECC during primary dentition, and to determine the optimal level for the prevention of ECC.

## Material and Methods

### Protocol Registration and Focused Question

This systematic review conformed to the PRISMA statement (registration number: CRD42022307655 under PROSPERO). The research question was determined following the PECO strategy: Population – preschool children; Exposure – diagnosis of ECC; Comparison – preschoolers without ECC; and Outcome – level of serum 25-hydroxyvitamin D. The following research question was established: Do preschool children with ECC have a lower level of serum 25-hydroxyvitamin D?

### Inclusion and Exclusion Criteria

Observational studies were included in this study if they met the following criteria: (1) the relationship between ECC and vitamin D was examined; (2) the study population consisted of children ≤6 years old or preschool children; (3) the primary outcome investigated between ECC and caries-free groups were the level of serum 25-hydroxyvitamin D, with means and standard deviation (SD); (4) laboratory assessment of prenatal or childhood vitamin D serum levels.

The exclusion criteria were: (1) no direct comparison between ECC and caries-free groups or no mention of vitamin D levels in ECC and caries-free groups; (2) incomplete data; (3) reviews, letters, abstracts.

### Search Strategy

Two researchers independently retrieved articles from December 2021 to February 2022, with no limit on language. The suitable searchwords were indexed in the electronic databases of PubMed, Web of Science, Cochrane and Embase. Moreover, additional records were identified through Google Scholar.

The MeSH terms used were “dental caries”, “child” and “vitamin D.” The following strategies were used to search in the PubMed: (((child[MeSH Terms]) OR (child[MeSH Terms])) AND ((((dental caries[MeSH Terms]) OR (dental caries)) OR (dental)) AND (caries))) AND ((vitamin D[MeSH Terms]) OR (vitamin D)). A similar search method was also applied in the other electronic databases, such as ((TS=(*vitamin D AND dental caries AND child) )) in Web of science, “vitamin D” and “caries” and “child” in Title Abstract Keyword in Cochrane library, and “dental caries”:ab,ti AND “vitamin D”:ab,ti in Embase. If additional data and articles were needed, we wrote e-mails to contact the relevant authors.

### Data Extraction and Quality Assessment

The following information from the included literature was collected and summarised by two researchers independently: first author, year of publication, study design, country, age, sample size of ECC and caries-free groups, the number of people with serum 25-hydroxyvitamin D levels > 75 nmol/l, = 50–75 nmol/l, < 50 nmol/l in the ECC and caries-free groups, the level of 25(OH)D with mean ± SD in the two groups. Disagreements were discussed with the senior authors and resolved by them. The Newcastle-Ottawa scale for cohort studies and case-control studies was employed, in addition to a modified version of the Newcastle-Ottawa scale for cross-sectional studies. Studies with seven or more points were considered to have high methodological quality. Studies with four to six points were considered to have medium methodological quality. Studies with three points were considered to have low methodological quality. According to the Newcastle-Ottawa scale rule, high-quality and medium-quality articles were included for analysis. Two reviewers assessed all these data, and dissent was settled by discussion or consultation with a third author.

### Statistical Analysis

The meta-analysis was performed using Review Manager 5.4 to analyse the correlation between vitamin D and ECC. A fixed-effects model was used for meta-analysis when the I^2^ statistic was < 50% or p > 0.10. If the result showed the high heterogeneity (I^2^ statistic > 50% or p < 0.10), a random-effects model was used for meta-analysis. The source of high heterogeneity was analysed through sensitivity analysis and subgroup analysis. If necessary, funnel plotting was used to analyse whether publication bias existed.

## Results

### Literature Search

**Fig 1:**
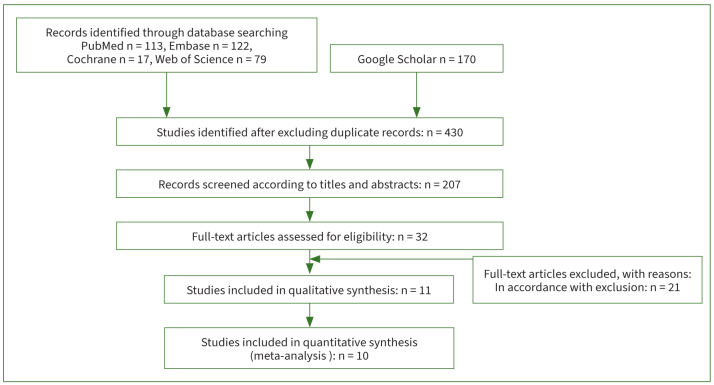
Flowchart of identified, included, and excluded studies.

Figure 1 illustrates the study selection process. A total of 501 articles were retrieved from the electronic databases. 71 duplicate articles in 501 articles were excluded. These 430 articles were screened based on the title and abstract, of which only 207 qualified for further retrieval. After a full-text search, only 32 articles proceeded to the next step. The next step was to remove articles meeting the following criteria: articles published in the form of letters, case reports, comments and conference abstract. Finally, 11 qualified articles were included in the systematic review, 10 of which were included for meta-analysis, i.e., 7 case-control studies, 3 cross-sectional studies and 1 cohort article.

### Study Characteristics

As shown in Table 1, the 11 studies were published between 2012 and 2021. Almost all of the studies were performed in Asia and North America, except the study by Andaur et al^8^ from Europe. Seven studies presented evidence that S-ECC is statistically significantly related to the status of vitamin D, with two of four articles reporting a statistically significant relation between ECC and vitamin D. The study by Seminario et al^42^ investigated the same sample of children in two different seasons and found that the level of serum 25-hydroxyvitamin D may vary from season to season, with summer being higher and winter lower. The vitamin D serum level in over half of the studies included were collected from May to October, so the levels of vitamin D measured in that period were utilised for the present meta-analysis. The findings of Hussein et al^25^ also identified no stastistically significant differences in serum 25(OH)D levels between children with and without caries experience. The status of vitamin D in serum was classified into three levels, as shown in six studies:^8,12,26,39,42,46^ ≥ 75 nmol/l, 50–75 nmol/l and < 50 nmol/l. Due to the different study designs and geographical regions, it was classified into subgroups for further analysis.

**Table 1:** Characteristics of the studies included

First author/ published year	Study design	Country	Age	N	ECC		Caries-free	25(OH)D (mean ± SD)
					25(OH)D (N)	25(OH)D (mean ± SD)	25(OH)D (N)	
Schroth et al, 2012	Case-control study	Canada	< 72 months	S-ECC: 19 Caries-free: 19	≥ 75 nmol/l: 2 < 75 nmol/l: 17	52.9 ± 15.1	≥ 75 nmol/l: 4 < 75 nmol/l: 15	64.4 ± 21.3
Schroth et al, 2013	Case-control study	Canada	≤ 71 months	S-ECC: 140 Caries-free: 121	≥ 75 nmol/l: 56 50–75 nmol/l: 55 < 50 nmol/l: 29	68.9 ± 27.9	≥ 75 nmol/l: 69 50–75 nmol/l: 38 < 50 nmol/l: 14	82.9 ± 31.1
Chhonkar et al, 2018	Case-control study	India	3–6 years old	S-ECC: 30 Caries-free: 30	NR	30.48 ± 10.9	NR	50.28 ± 10.3
Seminario et al, 2018	Cross-sectional study	USA	≤ 6 years old	ECC: 92 Caries-free: 184	≥ 75 nmol/l: 30 50–75 nmol/l: 46 < 50 nmol/l: 16	68.7 ± 28.1	≥ 75 nmol/l: 110 50–75 nmol/l: 55 < 50 nmol/l: 19	81.2 ± 38.7
Ahmed et al, 2020	Cross-sectional study	Iraq	2–6 years old	S-ECC: 47 Caries-free: 47	< 50 nmol/l: 39 ≥ 50nmol/l: 8	34.47 ± 14.2	< 50 nmol/l: 20 ≥50nmol/l: 20	51.27 ± 8.8
Jha et al, 2021	Case-Control study	India	40.82 ± 14.09 months	S-ECC: 140 Caries-free: 121	≥ 75 nmol/l: 56 50–75 nmol/l: 55 < 50 nmol/l: 29	68.89 ± 27.87	≥ 75 nmol/l: 69 50–75 nmol/l: 38 < 50 nmol/l: 14	82.91 ± 31.1
Williams et al, 2021	Case-Control study	Canada and the USA	< 72 months	S-ECC: 200 Caries-free: 144	≥ 75 nmol/l: 77 50–75 nmol/l: 83 < 50 nmol/l: 40	69.63 ± 30.94	≥ 75 nmol/l: 82 50–75 nmol/l: 49 < 50 nmol/l: 13	82.88 ±28.71
Andaur et al, 2021	Cohort study	Netherlands	6 years old	ECC: 1664 Caries-free: 3593	≥ 75 nmol/l: 387 50–75 nmol/l: 564 < 50 nmol/l: 713	NR	≥ 75 nmol/l: 1164 50–75nmol/l: 1325 < 50 nmol/l: 1104	NR
Chen et al, 2021	Cross-sectional study	China	24–72 months	ECC: 368 Caries-free: 1142	≥ 75 nmol/l: 111 50–75 nmol/l: 206 < 50 nmol/l: 51	67.45 ± 18.58	≥ 75 nmol/l: 433 50–75 nmol/l: 588 < 50 nmol/l: 121	70.88 ± 18.05
Ahmed S et al, 2021	Case-Control study	Saudi Arabia	5 years old	S-ECC: 300 Caries-free: 300	NR	30.48 ± 10.9	NR	50.28±10.3
Hussein et al, 2021	Case-Control study	Malaysia	<72 months	ECC: 93 Caries-free: 27	≥ 50 nmol/l: 42 < 50 nmol/l: 51	NR	≥ 50 nmol/l: 12 < 50 nmol/l: 15	NR

NR = not reported; ECC = early childhood caries; S-ECC = severe early childhood caries.

### Risk of Bias Appraisal

Tables 2 to 4 show the results of the quality appraisal of the articles. Four studies, Schroth et al,^38,39^ Williams et al^46^ and Andaur et al,^8^ received high-quality scores. The others were given medium-quality scores.

**Table 2:** Quality assessment of the cross-sectional studies included according to the modified Newcastle-Ottawa Scale^ǂ^

Author, year	Selection				Comparability	Results		Score
	Representativeness of the sample^[1]^	Sample size^[2]^	Ascertainment of the exposure^[3]^	Non-respondent^[4]^	Adjustment of confusion^[5]^	Assessment of outcome^[6]^	Statistical test^[7]^	
Seminario et al, 2018	(c)	*	*	*	–	*	*	5(8)
Ahmed et al, 2020	(c)	*	*	*	–	*	*	5(8)
Chen et al, 2021	*	*	*	*	–	*	*	6(8)

^ǂ^The following Newcastle-Ottawa Scale descriptions are explained in detail at https://www.ohri.ca/programs/clinical_epidemiology/oxford.asp. [1] (a) Truly representative of the average in the target population * (all subjects or random sampling); (b) somewhat representative of the average in the target population * (non-random sampling); (c) selected group of users; (d) no description of the sampling strategy. [2] (a) Justified and satisfactory*; (b) not justified. [3] (a) Validated measurement tool*; (b) non-validated measurement tool, but the tool is available or described *; (c) no description of the measurement tool. [4] (a) Comparability between the characteristics of respondents and non-respondents is established, and the response rate is satisfactory*; (b) the response rate is unsatisfactory, or the comparability between respondents and non-respondents is unsatisfactory; (c) no description of the response rate or the characteristics of responders and non-responders. [5] (a) The study controls for the most important factor (socioeconomic status)*; b) the study controls for any additional factor (age, household income, brushing habits, oral cognition level of parents)*. [6] (a) Independent blind assessment*; (b) record linkage*; (c) self-report; (d) no description. [7] (a)The statistical test used to analyze the data is clearly described and appropriate, and the measurement of the association is presented, including confidence intervals and the probability level (p-value)*; (b) the statistical test is not appropriate, not described or incomplete.

**Table 3:** Quality assessment of case-control studies included according to the Newcastle-Ottawa Scale^ǂ^

Author, year	Selection				Comparability	Results			Score
	Adequacy of case definition^[1]^	Selection of the non exposed case^[2]^	Selection of the control^[3]^	Definition of control^[4]^	Comparability of case and control on the basis of design or analysis^[5]^	Assessment of exposure ^[6]^	Same method of ascertainment for cases and controls ^[7]^	Non-Response rate ^[8]^	
Schroth et al, 2012	*	(b)	*	(b)	*	**	*	*	7(10)
Schroth et al, 2013	*	(b)	*	(b)	*	**	*	*	7(10)
Chhonkar et al, 2018	*	(b)	(b)	*	-	*	*	*	5(10)
Jha et al, 2021	*	(b)	*	(b)	-	*	*	*	5(10)
Williams et al, 2021	*	*	*	(b)	*	**	*	*	8(10)
Ahmed S et al, 2021	*	(b)	(b)	*	-	*	*	*	5(10)
Hussein et al, 2021	*	(b)	(b)	*	-	*	*	*	5(10)

^ǂ^The following Newcastle-Ottawa Scale descriptions are explained in detail at https://www.ohri.ca/programs/clinical_epidemiology/oxford.asp. [1] (a) with independent validation*; (b) record linkage or based on self-reports; (c) no description; [2] (a) consecutive or obviously representative series of cases*; (b) potential for selection biases or not stated; [3] (a) community controls*; (b) hospital controls; (c) no description; [4] (a) no history of disease (endpoint)*; (b) no description of source; [5] (a) The study controls for the most important factor (socioeconomic status)*; b) the study controls for any additional factor ( age, household income, brushing habits, oral cognition level of parents)*. [6] (a) secure record (e.g., surgical records) *; (b) structured interview with blinding to case/control status* (c) interviewer not blinded to case/control status; (d) written self-report or medical record only; (e) no description; [7] (a) yes *; (b) no; [8] (a) same rate for both groups*; (b) non-respondents described; (c) rate different and no designation.

**Table 4:** Quality assessment of the cohort studies included according to the Newcastle-Ottawa Scale^ǂ^

Author, year	Selection				Comparability	Results			Score
	Representativeness of the exposed cohort^[1]^	Selection of the non exposed cohort^[2]^	Ascertainment of the exposure^[3]^	Outcome not present at the start^[4]^	Comparability of cohorts on the basis of design or analysis^[5]^	Assessment of outcome ^[6]^	Follow-up time ^[7]^	Accuracy of follow-upt ^[8]^	
Andaur et al, 2021	*	*	*	*	*	*	*	*	8(9)

^ǂ^The following Newcastle-Ottawa Scale descriptions are explained in detail at https://www.ohri.ca/programs/clinical_epidemiology/oxford.asp. [1] (a) Truly representative of the average in the community*; (b) somewhat representative of the average in the community*; (c) selected group of users; (d) no description of the derivation of the cohort. [2] (a) Drawn from the same community as the exposed cohort*; (b) drawn from a different source; (c) no description of the derivation of the non-exposed cohort. [3] (a) Reliable record*; (b) structured interview*; (c) written self-report; (d) no description. [4] (a) Yes*; (b) no. [5] (a) Study controls for the most important factor (socioeconomic factors)*; (b) study controls for any additional factor* (age, household income,brushing habits, oral cognition level of parents). [6] (a) Independent blind assessment*; (b) record linkage*; (c) self report; (d) no description. [7] (a) Yes (≥12 months)*; (b) no (<12 months). [8] (a) Complete follow-up*; (b) subjects lost to follow-up unlikely to introduce bias (≥80 %)*; (c) follow-up rate <80% and no description of those lost; (d) no statement.

### Vitamin D Comparison Between ECC and Caries-free Groups

**Fig 2:**
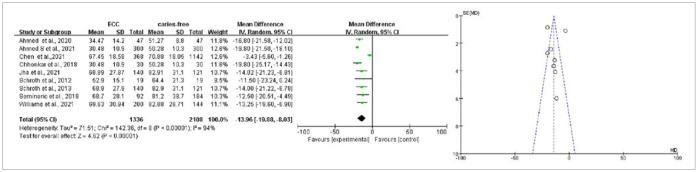
Forest and funnel plots of vitamin D comparison between ECC and caries-free groups.

**Fig 3:**
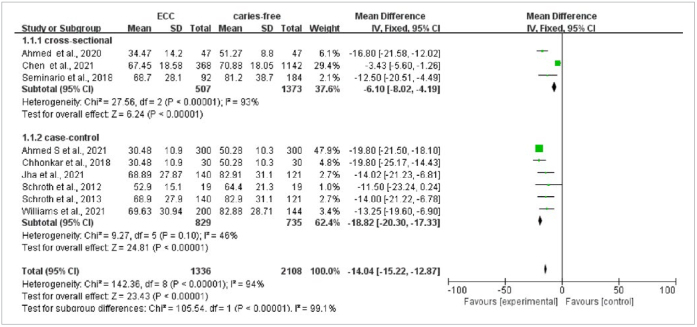
Vitamin D comparison between ECC and caries-free groups with subgroup analysis of geographical region.

**Fig 4:**
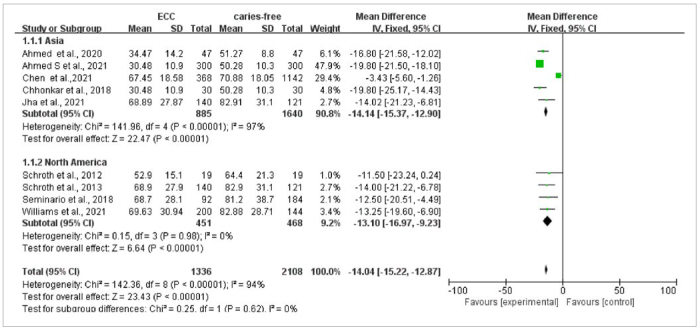
Vitamin D comparison between ECC and caries-free groups with subgroup analysis of geographical region.

Nine articles carried out a comparison of mean values of vitamin D in ECC and caries-free groups, including two articles on ECC and seven articles about S-ECC. The heterogeneity was statistically significant with p < 0.001, I^2^ = 94%, and a random-effects model was used for meta-analysis. The results of the meta-ana-lysis suggested that the 25(OH)D levels in the ECC group were statistically significantly lower vs those in the caries-free group (weighted mean difference [WMD] = -13.96, 95% CI: [-19.88, -8.03], p < 0.001); the funnel plot was basically symmetrical (Fig 2). In order to increase the robustness of the results and reduce inter-study heterogeneity, subgroups of study design and region were analysed. The outcome did not find the source of heterogeneity (Figs 3 and 4). The sensitivity analyses also were done by excluding one study each time, but the result was meaningless.

### Comparison of Vitamin D Levels between S-ECC and Caries-free Groups

**Fig 5:**
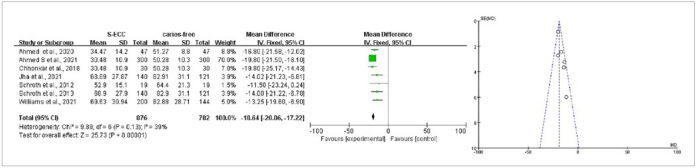
Vitamin D comparison between S-ECC and caries-free groups with funnel and forest plots.

**Fig 6:**
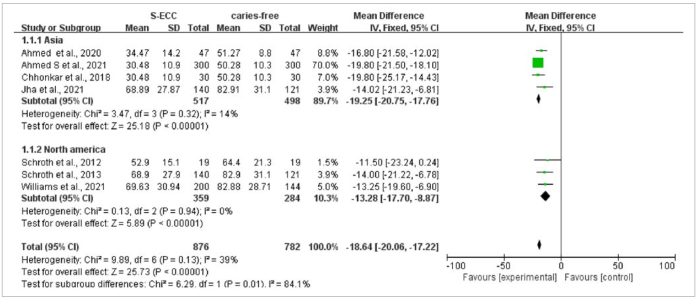
Vitamin D comparison between S-ECC and caries-free groups with subgroup analysis of geographical region.

Seven articles studied the relationship between mean vitamin D level and S-ECC. The heterogeneity was p = 0.13, I^2^ = 39%, and a fixed-effects model was used for meta-analysis. The results of the meta-analysis suggested that the 25(OH)D levels in the S-ECC group were statistically significantly lower compared with those of the caries-free group (WMD = -18.64, 95% CI: [-20.06,-17.22], p < 0.001), with the funnel plot basically symmetrical (Fig 5). The children with S-ECC from Asia had lower levelv of 25(OH)D than those in the caries-free group (WMD = -19.25, 95% CI: [-20.75,-17.76, p < 0.001). In North America, the 25(OH)D levels in the S-ECC group were statistically significantly lower compared with those of the caries-free group (WMD = -13.28, 95% CI: [-17.70,-8.87], p < 0.001). The subgroups analysis showed that different geographical regions were the main source of heterogeneity (p = 0. 01) (Fig 6).

### Rate of ECC with Different Levels of 25(OH)D

**Fig 7:**
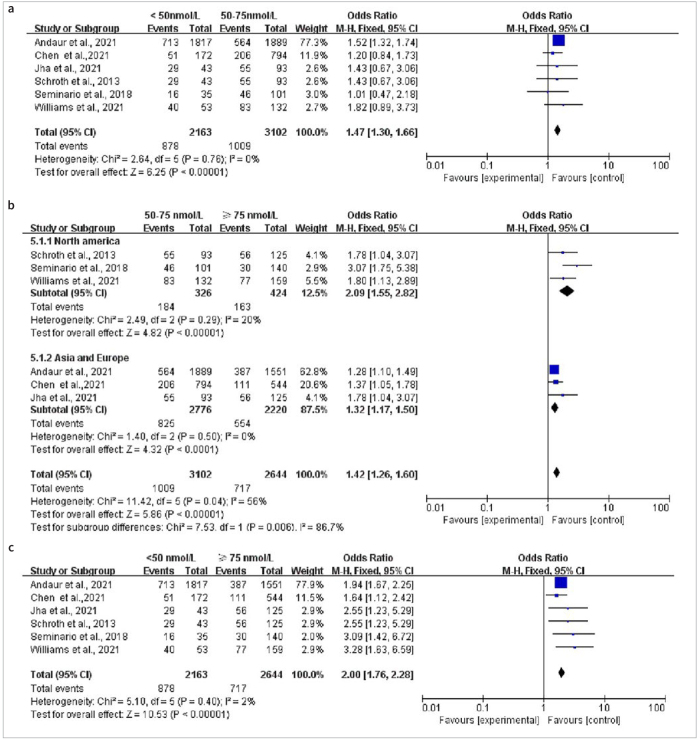
Forest plot of the proportion of ECC given different levels of 25(OH)D.

Six articles studied the proportion of ECC under different levels of 25(OH)D ranging from ≥ 75 nmol/l, 50–75 nmol/l to < 50 nmol/l. Statistically significant differences were found between the groups with 25(OH)D < 50 nmol/l vs the groups with 50–75 nmol/l (OR = 1.47, 95% CI: [1.30,1.66], p < 0.001) and over 75 nmol/l (OR = 2.00, 95% CI: [1.76,2.28], p < 0.001). The heterogeneity shown in Figs 7a and 7b was very low, while the heterogeneity shown in Fig 7c was high. The subgroup analyses in terms of geographical region demonstrated that children with 25(OH)D levels of 50–75 nmol/l were more likely to have ECC than those with > 75 nmol/l (OR = 1.42, 95% CI: [1.26,1.60], p < 0.001), with data from Asia and Europe combined for analysis. The subgroup analysis showed regional difference to be the main source of heterogeneity (p = 0.006).

## Discussion

This systematic review and meta-analysis used data of 8821 children to investigate the association between ECC and vitamin D with strict inclusion and exclusion criteria. The results of meta-analysis illustrated that statistically significant correlation was discovered for primary teeth.

There are several possible mechanisms by which vitamin D acts as a protecting factor in decreasing the occurrence and development of ECC. For instance, the function of vitamin D could maintain calcium and phosphate homeostasis essential to formation, calcification, mineralisation of teeth.^40^ When the homeostasis of calcium and phosphate is out of balance, hypocalcemia and hypophosphatemia occurs,^3^ leading to enamel and dentin defects. Another possible mechanism may be that the active form of vitamin D (1,25-dihydroxyvitamin D3) act directly on target cells of ameloblasts and odontoblasts.^3^ Vitamin D deficiency during tooth development may give rise to hypoplasia or hypomineralisation, leaving teeth susceptible to ECC.^48^ Further, vitamin D could induce certain antimicrobial peptides,^14,19^ which prevent the intrusion of and colonisation with pathogens.

However, the sensitivity and subgroup analyses could not identify the source of high heterogeneity in the relation between ECC and vitamin D. This may be due to the fact that ECC includes both ECC and S-ECC, and the latter is the more severe degree of decay. In addition, the factors associated with ECC were complicated, including social, biological and behavioural risk factors.^44^

It is of interest that the 25(OH)D levels in the S-ECC group were statistically significantly lower than those in the caries-free group with low heterogeneity. The cause may be related to developmental defects of primary teeth in a state of vitamin D deficiency, resulting in caries reaching more severe stages. The subgroup analysis demonstrated the 25(OH)D levels from Asia were statistically significantly different from those North America given the presence of S-ECC. The difference of skin colour is recognised as affecting vitamin D levels between Asia and North America, with a higher the utilisation rate of ultraviolet B in producing vitamin D in fairer-skinned subjects.^31^ Socioeconomic factors may be an important reason for the heterogeneity of the articles.^44^ Low socioeconomic status and high frequency of vegetarian diet are potential factors that lead to insufficient vitamin D intake among Indian children.^3^ However, direct evidence of such risks is still lacking.

There were statistically significant differences in the prevalence of ECC given < 50 nmol/l, 50–75 nmol/l and ≥ 75 nmol/l of 25(OH)D levels. With increased 25(OH)D levels, the risk of suffering from ECC decreased gradually in our study. The 25(OH)D concentrations (≥ 75 nmol/l) is recognised as the optimal value in many studies.^10,46^ The First International Conference^41^ on vitamin D held a debate on feasible concentrations of 25(OH)D and came to the conclusion that levels over 30 nmol/l should be regarded as the lower limit for reducing the risk of rickets, whereas 25(OH)D levels between 50–125 nmol/l seem to be appropriate for skeletal growth and oral health.^41^

The comparison between 50–75 nmol/l and ≥ 75 nmol/l showed higher heterogeneity than in other groups. The subgroup analysis found the protective effect of 25(OH)D levels (≥ 75 nmol/l) to be stronger in North America than in Asia and Europe. As mentioned before, the 25(OH)D levels of Asia were statistically significantly lower than in North America. The intake of vitamin D is far below the recommendation of the European Academy of Paediatrics, as not all European nations fortify foods with vitamin D.^23^ Depending on the country, vitamin D deficiency is variously emphasised. For instance, the US Institute of Medicine and the Endocrine Society of Canada have advocated the utilisation of vitamin D-fortified foods for general health for many years.^11^

Some studies^3,27^ found common risk factors between ECC and vitamin deficiency, including increase age, obesity, low socioeconomic status and systemic diseases such as iron deficiency anemia.

The difference in methodological quality is mainly due to the fact that some articles did not control for socioeconomic status. This discrepancy may indicate that socioeconomic status is a potential epidemiological risk factor for ECC. Socioeconomic differences between regions may potentially affect the reported frequencies of ECC.

The prevalence of vitamin D deficiency increases with age in preschool children, yet data on the mechanisms involved are still inconclusive.^3^ ECC as a dynamic progression of disease also increases with age. The review about the association between ECC and vitamin D status found that low socioeconomic status and obesity were risk factors influencing vitamin D status.^3^ Some studies have confirmed low socioeconomic status as mutual risk factor of vitamin D deficiency and ECC,^39,46^ in which children have inadequate nutrition, leading to a higher prevalence of ECC.^7^ Another factor that may contribute to both ECC and low vitamin D intake is obesity, in which vitamin D accumulates in the body’s adipose tissue and alters the expression levels of vitamin D-metabolizing enzymes.^36^ It is unlikely that obese children actively participate in outdoor exercise,^43^ inhibiting the production of this vitamin. Based on recent evidence about the association between obesity and ECC as shown in a meta-analysis,^33^ the specific mechanism of this association is inconclusive. After controlling for the influence of potential confounding factors, the socioeconomic status and parental education levels may be important elements.^12^

When iron deficiency anemia, vitamin D deficiency, and ECC were studied together,^17^ the results showed that children with ECC seem to be at a significantly greater risk of vitamin D deficiency and iron deficiency anemia. However, the phenomenon may be coincidental. Healthy children may also have a high incidence of combined deficiencies of 25(OH) D and iron. Over half the children with iron deficiency anemia or iron deficiency also had vitamin D deficiency, when compared with 29% of normal controls in the study.^28^ Another study demonstrated that a statistically significant number of children with vitamin D deficiency also suffered from iron deficiency anemia.^47^ Theoretically, vitamin D metabolism may play a role in erythropoiesis, while iron-dependent enzymes are required for activation of vitamin D metabolites.^6,30^ Further research is needed.

### Limitations

The type of included studies was non-uniform, involving in cross-sectional, case-control and cohort studies. The cross-sectional design was not appropriate for determining a causal relationship, but only supported an association between ECC and vitamin D, due to the lack of prior data on children’s vitamin D status and tooth development. The cohort study by Andaur et al^8^ investigated the associations between prenatal, perinatal, and early childhood vitamin D status and risk of dental caries at 6 years. Only their early childhood data was further analysed in our study, so that a meta-analysis of cohort studies is necessary as a more rational method for further analysis of the relation between vitamin D and ECC.

Another limitation is that ECC criteria are based on the dmft index, as opposed to the ICDAS II, in which lesions are classified into cavitated and non-cavitated, a more complex method that is better for detecting the early phase of carious lesions.

## Conclusion

The cross-sectional and case-control study testified that the vitamin D deficiency was related to ECC, so it would be more credible to carry out a cohort study about the caries experience between a control group and vitamin D deficiency groups.

In the future, a more standardised clinical experimental design and cohort study design would help to reveal the relationship between vitamin D and ECC and provide useful approaches to clinical prevention. The level of vitamin D was lower in children with ECC than caries-free children, and this correlation between S-ECC and vitamin D was even more pronounced. The optimal 25(OH)D level for preventing occurrence and development of ECC was ≥ 75 nmol/l. Thus, clinicians should view the development of early childhood caries also from a systemic perspective.
